# Diphtheria: Remarks on the Germ Theory, with Report of Cases under Alcoholic Treatment*Read before the Chicago Medical Society, January 5, 1885.

**Published:** 1885-02

**Authors:** G. H. Chapman

**Affiliations:** 7725 Greenwood Ave., Grand Crossing, Ill.


### ARTICLE IV.

DIPHTHERIA: *Remarks on the Germ Theory, with Report of Cases under Alcoholic Treatment.** *By* G. H. CHAPMAN, M. D.

There is perhaps no disease known to medical science which has been so constantly and thoroughly discussed in all its bearings, as to its origin, history, pathology, and treatment, as diphtheria, and still leaves us so comparatively in the dark as to reliable remedies. I think I would not go astray in saying that every physician, the veteran, as well as those who are just entering the field, is ready to adopt any method of treatment which offers a reasonable promise of securing the end sought, viz.: the cure of the patient.

It is not my intention to enter into this subject in detail in this paper, but simply to present to your minds a few facts which have, in my experience, proved of value in combating this dread disease.

And first as to its development. Is it local, or does it involve the whole system? Many writers have considered it a purely local disease, and as such they advocated only local remedies, while others have gone to the other extreme and given no attention to local measures. Perhaps the most recent and universal theory has been that given by Oertel, that diphtheria begins as a local disease and develops afterwards into a general one; and that, moreover, the general infection is kept up by the local one. The disease germ attaches itself to the laryngeal mucous membrane, from which point by its rapid reproduction the whole system becomes speedily infected and the micrococci can be found in great numbers in all the tissues and fluids of the body.

Dr. H. C. Wood, in speaking of this matter before the Penn-

---

*Read before the Chicago Medical Society, January 5, 1885.

sylvania State Medical Association, last May, said, that Dr. Henry Formad and himself had been occupying their leisure time during the past three or four years investigating the subject of diphtheria; and when the attempt was made to infect the lower animals by using the material as obtained from an ordinary type of diphtheria as it appeared in Philadelphia, they failed; but when taken from those who were subjects of a malignant epidemic they invariably succeeded in infecting the lower animals; he also said it could be communicated from animals to man, the human subject.

They procured diphtheritic membrane, soaked it in water, then filtered it, and found the filtrate innocuous; while the residue was very poisonous and capable of producing diphtheria.

They also procured the membrane and cultivated the micrococci in soups, and found that they produced diphtheria with micrococci of the fourth and fifth generation; at least they were unable to distinguish the disease thus produced from diphtheria. Here, however, is an objection. They found in *ordinary saliva* a micrococcus, or plant, which cannot be distinguished from the micrococcus of the most malignant diphtheria; they were, as far as could be discovered, identical, and in everybody's mouth. They further noted a grade in the poisoning power. The micrococcus from a sound throat produced no poisoning, and the severity of the poison was directly as the virulence of the epidemic from which the poison was taken. Dr. Wood thinks that these researches prove that diphtheria is produced by micrococci that are present in every mouth.

They also conclude that in the majority of cases the disease was first local, the micrococci attaching themselves to the mucous membrane where they multiply and produce surrounding inflammation, the "membrane becoming filled with micrococci they then force their way between the epithelial cells, the cells begin to die, and ulceration takes place; the mouths of the lymphatics open and the micrococci pass in. Early in the disease no micrococci are found in the blood, but after the constitutional symptoms have become severe, masses of them will be found in the vessels, and the plant invades the white blood corpuscles, which after a time will be found filled with micrococci; they will be found in the kidneys, in the capillaries, forcing their way between the muscular fibres of the heart and all over the body leading everywhere to destruction of tissue. Matter has also been taken from the discharge of puerperal metritis and from sloughing gangrenous ulcers and found full of microbes, which upon being inserted into animals produced a disease which could not be distinguished from diphtheria."

These are the conclusions of those who have spent several years in the scientific investigation of the subject, with abundance of material at their disposal, and would it not seem to disapprove the germ theory of the disease? We would not dispute the fact that microbes are in all diphtheritic membrane, but they are also present where there is no diphtheria, in sound throats as well as in septic discharges from other diseases, and are not found in the blood of diphtheritic patients "till constitutional symptoms have become severe."

If, as they affirm, "micrococci can be found in ordinary saliva which in no way differ from those found in malignant diphtheritic membrane," except possibly that they are inert, even though they may only be found in the saliva of those who frequent or inhabit the infected district, it would seem to prove that they alone are not responsible for the development of diphtheria; there must be *conditions for* their rapid reproduction which are not yet understood, and may it not be possible that they simply find in this degenerated or septic condition of the fluids and tissues a good field for growth and development in an active state, and still not be the primary cause of diphtheria? Be this disease, however, purely local or general in its origin, be there micrococci or no micrococci as a primary cause for its development, the stubborn fact remains that in nine cases out of ten when the physician is first summoned to attend a case of diphtheria, he finds abundant evidence of general systemic infection, varying in degrees of intensity from simple circulatory excitement to the most profound degree of prostration found in septicæmia, and as such we are obliged to meet it.

A very severe form of diphtheria manifested itself in our community during the fall and winter of 1881 and 1882, commencing about the middle of September and continuing severe till about the middle of January, 1882. During the first six weeks of the epidemic I attended seven cases, four of which were mild in character, and readily yielded to ordinary treatment, the other three presented malignant characteristics, one died within twenty-four hours with croup as a complication, one died on the third day, and the third recovered after a protracted and debilitating illness.

My treatment in these cases was extremely free use of camphorated oil and turpentine, and application to the diphtheritic membrane every four or six hours with a camel's hair brush the following:

℞ Acid. Carbolici.................................f. ℥i
Liq. Ferri Subsulph. .........................f. ℥iii
Glycerin. ...........................................f. ℥iv
M.

and during the interval directed frequent gargling with a saturated aqueous solution of chlorate of potash. Internally administered the following:

℞ Tr. Ferri Hydrochlor.... ................f. ℥iii
Quiniæ Sulph. ...............................f. ℥i
Potass. Chlor. ................................f. ℥i
Syr. Glycyrrhiz. .............................f. ℥iiiss
M.

in doses to suit the age of the child, giving to a child of eight years one teaspoonful every three hours. This formed the basis of treatment, other remedies, diuretic, laxative, etc., being used as each individual case seemed to demand.

In the case which seemed malignant and recovered, powdered sulphur was used after the fourth day, in the throat and nasal cavities, being blown in through a goose quill every six hours, after cleansing with the chlorate of potash gargle.

There had been up to this time (Nov. 1) about twenty cases in our immediate vicinity with twelve deaths, other physicians, local and foreign, succeeding no better than I in controlling the disease.

My attention was called to a short article in the Oct., 1881, number of the *Boston Journal of Chemistry*, where a physician who had diphtheria himself, took by mistake a swallow of dilute alcohol, and was surprised when he recovered from the strangling to find that he had coughed up large masses of membrane and felt relieved. He continued its use in his own case and subsequently in his practice with good success. I also recalled an article in the *Eclectic Medical Journal* of Cincinnati, March, 1881, where the writer, after referring to the analogy of the diphtheritic fungi to that found upon stale bread, belonging to the family of the *penicillium glaucum*, quotes from "Raue's Pathology and Therapeutic Hints," as follows:

"Pieces of bread were taken, covered all over with a dense vegetation of *penicillium glaucum.* Then he poured upon one a concentrated solution of nitrate of silver; upon another a solution of chlorate of lime; upon the third, a solution of caustic potash; upon a fourth, a solution of sulphate of copper; upon a fifth, chloride of iron; upon a sixth, corrosive sublimate; upon a seventh, spirits of camphor; and upon the last, alcohol. The alcohol at once caused all the fungous growth to be thrown down and totally destroyed; so did the spirits of camphor—converting them at the same time into an amorphous mass; but all the other substances did not seem to affect the fungus growth in the least."

Even though the diphtheritic fungi be of a specific nature and essentially different from those found upon mouldy bread, an opinion which it is natural for us to entertain, still the theory of applying alcohol for its destruction would remain the same.

Interesting experiments with diphtheritic membrane are also detailed in *Ziemmssen's Cyclopædia*, vol. 1, page 680, from which we infer that alcohol is the safest agent which can be used to destroy or render inert these micrococci. I say safest, because other agents which will destoy their activity are liable to produce injury to the patient if not used by skilled hands, and alcohol can only intoxicate, and in diphtheria seldom does that, even if used in great excess.

I determined to give a fair trial to the alcoholic treatment, and use dilute alcohol with chlorate of potash as a gargle, powdered sulphur dry in the throat, and the internal use of hot whisky sling or milk punch, strong as could be borne, and only to be limited by the amount which would be tolerated without producing intoxication, with other remedies, as the cases might require to maintain the proper secretions of the body.

As a result of this trial I can report that my succeeding twenty-eight cases were carried to a successful termination, with one exception; and in none of them was there used a brush or probang to the throat.

The details as to condition, symptoms and treatment of a few cases are here given.

*Case VIII.*—(First under alcoholic treatment.) B. J., aged 13, strong, muscular, healthy boy, was first taken sick at noon Oct. 31. Had been feeling usually well for the preceding few days and up to the time of attack, when he felt some nausea, backache, and had a severe chill. I saw him first at 7 P. M., and found him with a dark flushed face, skin hot and dry; complained of severe headache and pain in left tonsil, which was tender on pressure from outside, breath very offensive, tongue coated, edges purplish in color. Tonsils enlarged, with a depressed mucous patch on the left one, about three lines in diameter. This tonsil had a purplish appearance, which grew darker as it approached the edge of the ulcer, and this ulcer a greenish, dirty white color. Some coryza and obstruction to nasal passages.

Temp., 105°; pulse, 120, full and bounding; patient quite restless and at times delirious.

*Diagnosis—Diphtheria.*—Treatment: Locally, apply over each tonsil a cloth saturated with carbon oil, and as soon as the skin shows irritation, apply a thin slice of salt fat pork covered with pepper.

As a gargle:

℞. Alcohol....................................f. ℥viii
Chlorate of potash.........................f. ℥iii
Water........................................f. ℥viii
M.

and gargle freely every half hour, and wash the mouth with the same before swallowing either medicine, food or drink; also, every four hours, blow powdered sulphur into the throat after using gargle.

Internally:

℞ Tr. verat. virid.

Tr. opii deodorat. ........................ āā, f. ʒss

Aquæ ........................................ f. ℥iv

M.

one teaspoonful every two hours till sweating occurs or fever abates; an improved compound cathartic pill to be taken at nine o'clock.

Nov. 1, 7 A. M. No change in the condition except a spreading of the membrane on the left tonsil to about six lines in diameter, and a spot about three lines in diameter on the right tonsil, with a slight decrease of the blue tinge of the mucous membrane. There being no good whisky at hand, I ordered a strong hot alcohol punch every two hours and continue other remedies as before.

12, noon. Decided diminution of fever; membrane appears shriveled with the edges rolling towards the center. Having now procured some good rye whisky, I ordered it given freely in the form of milk punch or sling, hot as it could be taken.

8 P. M. Membrane becoming rapidly detached from the left tonsil, and that on the right one presents the same shriveled appearance as was seen on the left one at noon.

The veratrum mixture was now discontinued, but otherwise treatment was the same.

Nov. 2, 7 A. M. Temp., 99; pulse, 90. Membrane all gone from left tonsil and nearly gone from the right one. Continue same treatment.

8 P. M. Temp. normal. Tonsils present a healthy pink color, with no membrane visible, and this in forty-eight hours from time of commencement of treatment. I visited the patient the following day and then dismissed the case.

*Case 9.*—Carl E., age 7. Was called at 5 A. M., November 1st, to see him, and found him with temp. 105°, pulse 140, and delirious, face flushed, and the condition of tonsils and surrounding tissues the same as in the previous case. I ordered the veratrum and opium mixture every two hours, and all the hot, strong whisky sling, or milk punch, he could be induced to take, adding a little saturated solution of chlorate of potash to every wine-glass of punch given, with local use of oil and pork, as in previous case. During my temporary absence from town the parents became frightened and called in a brother physician whom I found there on my return at noon. He said: "Doctor, you have here a very malignant case of diphtheria, and unless you treat the throat locally and treat vigorously, you will lose the patient."

I protested against any change of treatment and continued with the case as I had commenced, crowding the whisky as much as possible. At 11 P. M. he broke out in warm perspiration, dropped into a quiet sleep, and from that moment began to improve.

On the morning of November 2d, the membrane had the same shriveled appearance as that described in the previous case, and at 7 P. M., thirty-eight hours after commencement of treatment, no vestige of membrane could be found on either tonsil. He recovered very rapidly and soon was out of my care, cured.

*Case XI* occurred in a child only thirteen months old. The condition seemed one of malignant character, as the others, and whisky was depended on as in the other cases, with the same results. This child took in thirty-six hours one pint of the best rye whisky without producing intoxication, but completely relieving the stupor in which the child was when treatment was commenced, and caused the membrane to shrivel up and become detached as in former cases.

These are typical of the severe cases treated during this epidemic, and all were treated in a similar manner, varying as the difference of age and surroundings demanded.

As before stated, only one case was lost of the twenty-eight treated by the alcoholic treatment. This occurred in a girl eleven years of age, of nervous temperament, who six months previous had had a severe attack of typhoid fever, from which she had not recovered her usual nervous energy; consequently, when attacked by diphtheria of a severe naso-pharyngeal character, it was difficult to maintain the strength to resist its ravages, and she died on the eighteenth day from paralysis of the heart.

During the month of July, 1882, I attended three cases of croupous diphtheria, two of which died on the third day. The third one was seen on the third day (having been under treatment two days), and it seemed impossible for life to be prolonged an hour. I, however, ordered:

℞ Hydrarg. chlor. cor. .......................... gr. ii.
Tr. Aconit. ................................ m. x.
Aquæ ........................................ f. ℥ viii.

M. et S.

one teaspoonful every hour. The milk punch and whisky sling to be continued as before. I left the house with instructions that I should be sent for if the child lived till night that I might advise as to farther treatment. The next morning being in the same neighborhood I dropped in and was surprised to find the child running around the floor, dragging a broom after itself in great glee.

Within the past two years I have used more constantly the liquor ferri subsulph. with carbolic acid and glycerine (applying it to the throat with a camel's hair throat brush) instead of sulphur. And I think the results have been full as satisfactory. My sheet anchor, however, is alcohol in some form in every stage of the disease. Veratrum virdide and opium in the congestive condition, ferruginous tonics during convalescence if required, counter-irritation over the tonsils and careful observance of all the surroundings of the patient as to temperature, ventilation, drafts of air, etc. Success in the treatment depends as much upon attention given to the surroundings of the patient as upon the details of medication.

With this line of treatment the cases are brought to the convalescent stage quicker and with less general debility than where severe caustics are used, and the severer forms of treatment.

7725 Greenwood Ave., Grand Crossing, Ill.

